# Transparent Poly(amide-imide)s with Low Coefficient of Thermal Expansion from Trifluoromethylated Trimellitic Anhydride

**DOI:** 10.3390/polym17030309

**Published:** 2025-01-24

**Authors:** Seong Jong Kim, SeongUk Jeong, Taejoon Byun, Jun Sung Kim, Haeshin Lee, Sang Youl Kim

**Affiliations:** Department of Chemistry, Korea Advanced Institute of Science and Technology (KAIST), Daejeon 34141, Republic of Korea; krisj@kaist.ac.kr (S.J.K.); andoraed@kaist.ac.kr (S.J.); ruminaire@kaist.ac.kr (T.B.); kjs9209747@kaist.ac.kr (J.S.K.)

**Keywords:** high-performance polymer, poly(amide-imide), transparent polymer film, flexible display

## Abstract

Making transparent aromatic polymers with high T_g_ and low thermal expansion behavior, like glass, is challenging. We report transparent and soluble poly(amide-imide)s (PAIs) with high dimensional stability synthesized from the new monomer, trifluoromethylated trimellitic anhydride. Insertion of trifluoromethyl (CF_3_) groups into polymer chains enhanced solubility and the optical properties of polymers without sacrificing high thermal stability. Model reactions were utilized to study how the CF_3_ group in trimellitic anhydride affects the polymerization reaction with aromatic diamine monomers, and a series of new PAIs were synthesized. All the polymers were soluble in polar organic solvents and can be solution-cast into nearly colorless and flexible freestanding films. The obtained PAI films possessed high thermal stability (*T*_d5_: 437–452 °C in N_2_) and high transparency (84~87% transmittance at 550 nm). Interestingly, PAIs prepared in this study exhibited high thermodimensional stability with low CTE values from 9 to 26 ppm/°C. The transparent poly(amide-imide) film with low CTE value finds its application in display and optical devices that require flexible and transparent form factors.

## 1. Introduction

Highly transparent and thermally stable polymer films have become key materials in fabricating flexible and wearable display devices by replacing glass substrates [1,2]. Though transparent commercial polymer films, such as poly(ethylene terephthalate) (PET) and poly(ethylene naphthalate) (PEN), show good optical properties, their thermal stability is not high enough to withstand the processing conditions of OLED display devices, requiring thermodimensional stability up to at least 350 °C [3,4]. For this reason, high-temperature polymers with good optical properties have emerged as substrate materials for flexible and transparent display devices [5], and polyimide (PI) has attracted significant attention among high-temperature polymers as its features are ideally suited to high-temperature operations [6]. Because of its highly planar chain structure and high stiffness, polyimides have a very high thermal decomposition temperature (*T*_d_), high glass transition temperature (*T*_g_), and low coefficient of thermal expansion (CTE) [7]. These excellent physical properties of polyimides are further fortified by the charge transfer (CT) interaction between polymer chains. When electron-rich amines interact with anhydrides that lack electrons, a CT complex is formed, resulting in tight polymer chain stacking with excellent thermal stability. However, because the CT complex brings a yellowish-to-brown color, enhancing the transparency of PIs without sacrificing their thermal properties is still challenging [8,9,10]. To improve the color and transparency problems of polyimides, many structural variations of polyimides have been attempted for transparent PIs without sacrificing their excellent properties. The successful approaches include the insertion of flexible or unsymmetrical linkages or bulky substituents on the main chain and the introduction of noncoplanar or alicyclic units [11,12,13,14,15,16,17,18,19,20]. However, many cases of structural modification of aromatic polyimides show contradictory results between color and other physical properties, including thermal stability, glass transition temperature, and coefficient of thermal expansion of polyimides [21,22,23,24,25,26,27]. For example, although the ether linkage has been generally recognized as a flexible linkage as it offers improved color and transparency, it decreases the T_g_ value and reduces the thermal stability of polyimides due to the increased segmental motion of the polymer chains. Among many structural variations of polyimides, inserting the trifluoromethyl (CF_3_) and aromatic amide groups into the polyimide chains is particularly effective in enhancing the thermodimensional and optical properties of polymers [28,29,30,31,32,33]. Bulky CF_3_ groups reduce CT complex formation by effectively preventing interchain packing by disrupting the overall planarity of the polymer chain, while hydrogen bonding of amide and CF_3_ groups increases the thermodimensional stability of polymer chains. Interestingly, the incorporation of two trifluoromethyl (CF_3_) groups at the ortho positions of the rigid biphenyl linkage into the polymers enhanced the transparency of polyimides without deterioration in the glass transition temperature (*T*_g_) or thermal stability [34,35,36]. The CF_3_ groups effectively hinder the rotation of the phenyl rings along the C-C bond and decrease the lowest unoccupied molecular orbital level of the nitrogen atom to prevent the formation of charge transfer complexes [37,38]. F-H hydrogen bonding also can contribute to interchain interactions [39].

In this research, we designed and synthesized thermally stable and transparent PAIs from trimellitic anhydride substituted with a CF_3_ group. We devised a method for synthesizing the trifluoromethylated trimellitic anhydride monomer and polymerized it with a diamine having a biphenyl group with two CF_3_ groups to maximize transparency. Model reactions were also carried out to study how the CF_3_ substituent of the trimellitic anhydride monomer affects the polymerization reaction. The effect of the trifluoromethyl group and hydrogen bonding in the transparency and thermodimensional stability of aromatic polymers makes the new trifluoromethylated trimellitic acid–anhydride monomer synthesized in this study very versatile for making high-performance polymers with transparency and high thermal and dimensional stability which find applications in transparent display and optical devices.

## 2. Materials and Methods

### 2.1. Materials

5-Bromo-1,2,4-trimethylbenzene (Alfa Aesar, Ward Hill, MA, USA, 99%), sodium hydroxide (NaOH, Daejung, 99%), potassium permanganate (KMnO_4_, Aldrich, Saint Louis, MO, USA, 99%), methanol (Daejung, Seoul, Republic of Korea, 99.8%), methyldifluoro(fluorosulfonyl)acetate (FSO_2_CF_2_COOMe, TCI, Tokyo, Japan, 97%), tris(dibenzylideneacetone)dipalladium(0)-chloroform adduct (Pd_2_(dba)_3_-CHCl_3_, Aldrich, 98%), triphenylarsine (Aldrich, 97%), copper (I) iodide (Aldrich, 99.5%), 1,4-diazabicyclo [2.2.2]octane (DABCO, Aldrich, 99%), 4-aminobenzoic acid (Aldrich, 99%), 3-aminobenzotrifluoride (TCI, 99%), *p*-anisidine (Aldrich, 99%), anhydrous 1,4-dioxane (Aldrich, Saint Louis, MO, USA, 99.8%), acetic anhydride (Aldrich, Saint Louis, MO, USA, 99.5%), anhydrous N,N-dimethylformamide (DMF, Aldrich, Saint Louis, MO, USA, 99.8%), anhydrous N,N-dimethylacetamide (DMAc, Aldrich, Saint Louis, MO, USA, 99.8%), anhydrous N-methyl-2-pyrrolidone (NMP, Aldrich, Saint Louis, MO, USA, 99.5%), anhydrous pyridine (Aldrich, Saint Louis, MO, USA, 99.8%), and triphenyl phosphite (TCI, Tokyo, Japan, 97%) were purchased from commercial vendors and used as received. 2,2′-Bis(trifluoromethyl)benzidine (***s*DA**) was purchased from TCI and purified by vacuum sublimation at 120 °C. Terephthalic acid (**TA**) was purchased from TCI and purified by vacuum sublimation at 210 °C. 2,2′-Bis(trifluoromethyl)-4,4′-bis(trimellitimido)biphenyl (***s*DAc**) was synthesized as previously reported [30]. Calcium chloride (CaCl_2_) was purchased from Junsei and dried overnight under vacuum at 180 °C prior to use. All other commercially available reagent-grade chemicals were used without further purification.

### 2.2. Methods

The NMR spectra of the synthesized compounds were recorded on a Bruker Fourier Transform Advance 400 spectrometer. The chemical shift in the NMR was reported in parts per million (ppm) using tetramethylsilane as an internal reference. Splitting patterns were designated as s (singlet), d (doublet), dd (doublets of doublet), t (triplet), q (quartet), or m (multiplet). Elemental analyses (EAs) of the synthesized compounds were carried out with a FLASH 2000 series. The inherent viscosity of the synthesized polymers was measured using an Ubbelohde viscometer at 30 °C. The sample concentration was 0.5 g/dL in DMAc. The Fourier-transform infrared (FT-IR) spectra of the compounds were obtained with a Thermo Fisher Scientific Nicolet iS50 FTIR spectrophotometer (Waltham, MA, USA) through the attenuated total reflection (ATR) technique (number of scans: 16; resolution: 0.4 cm^−1^). The UV–visible spectra were obtained from a Shimadzu UV-2600 spectrometer (Kyoto, Japan) in transmittance mode. The thickness of the film for UV–visible analysis was 30 μm. Thermogravimetric analysis (TGA) and differential scanning calorimetry (DSC) were performed on a TA Instruments TGA Q50 and a DSC Q20 instrument, respectively. The TGA measurements were conducted at a heating rate of 10 °C/min in N_2_ and air. The glass transition temperature (*T*_g_) values of the polymers were obtained with a DSC instrument at a heating rate of 10 °C/min in N_2_. Samples of 10 mg were used. The in-plane linear coefficients of thermal expansion (CTEs) of polymer films were measured by thermal mechanical analysis (TMA) using a TA TMA-Q400 thermomechanical analyzer (New Castle, DE, USA) The CTE value was calculated using the temperature range of 50 °C to 250 °C in second and third heating runs, respectively. The UV–visible spectra were obtained from a Shimadzu UV-2600 spectrometer in transmittance mode.

### 2.3. Monomer Synthesis

**5-bromobenzene-1,2,4-tricarboxylic acid (1)**: A 500 mL two-neck round-bottom flask was charged with 5-bromo-1,2,4-trimethylbenzene (12.00 g, 60.3 mmol), NaOH (3.00 g, 75.0 mmol), KMnO_4_ (63.00 g, 399.0 mmol), and 30 mL of water. The mixture was gradually heated to 90 °C for 30 min and 100 °C for 30 min and refluxed for 24 h. The solution was hot-filtered to remove MnO_2_ and washed thoroughly with hot water. After the solution was acidified via concentrated HCl until reaching pH 1, the solution was extracted with diethyl ether, washed with brine, dried over MgSO_4_, and filtered. The solution was concentrated using a rotary evaporator and dried in a vacuum oven at 80 °C for 15 h to obtain the product as white powder **1** (5.35 g, 30.7% yield). ^1^H NMR (DMSO-*d*_6_, 400 MHz, ppm): 13.69 (broad, COOH, 3H), 8.02 (d, J = 0.4 Hz, 1H), 7.94 (d, J = 0.4 Hz, 1H).

**Trimethyl 5-bromobenzene-1,2,4-tricarboxylate (2)**: A 250 mL two-neck round-bottom flask was charged with **1** (3.00 g, 10.30 mmol), 80 mL of methanol, and 3 mL of conc. H_2_SO_4_. The mixture was heated up to 75 °C and refluxed for 18 h. The reaction mixture was extracted with diethyl ether, washed with brine, dried over MgSO_4_, and concentrated using a rotary evaporator. The product was purified via silica gel column chromatography using diethyl ether/hexane (*v*/*v* = 1:1.2) as an eluent. The product was dried in vacuo at r.t. for 2.5 h to give white solid **2** (3.26 g, 95.2% yield). ^1^H NMR (Chloroform-*d*, 400 MHz, ppm): 8.18 (s, 1H), 7.94 (s, 1H), 3.95 (s, 3H), 3.92 (s, 3H), 3.90 (s, 3H).

**Trimethyl 5-(trifluoromethyl)benzene-1,2,4-tricarboxylate (3)**: A 250 mL two-neck round-bottom flask was charged with **2** (3.00 g, 9.06 mmol), FSO_2_CF_2_COOMe (8.70 g, 45.30 mmol), Pd_2_(dba)_3_-CHCl_3_ (0.47 g, 0.45 mmol), triphenylarsine (1.11 g, 3.62 mmol), copper(Ⅰ) iodide (8.63 g, 45.30 mmol), and 75 mL of DMF. The reaction mixture was heated to 120 °C and stirred for 14 h. After cooling, the reaction mixture was diluted with diethyl ether and filtered through celite. The filtrate was washed three times with water, and the organic layer was dried with MgSO_4_. The resulting solution was filtered and concentrated by a rotary evaporator. The product was purified via silica gel column chromatography using diethyl ether/hexane (*v*/*v* = 1:1) as an eluent. The product was dried in vacuo at r.t. for 2.5 h to give white solid **3** (2.90 g, 100.0% yield). ^1^H NMR (DMSO-*d*_6_, 400 MHz, ppm): 8.21 (s, 2H), 3.91 (s, 3H), 3.88 (s, 6H).

**5-(trifluoromethyl)benzene-1,2,4-tricarboxylic acid (4)**: A 250 mL two-neck round-bottom flask was charged with **3** (2.90 g, 9.05 mmol), NaOH (12.00 g, 300.00 mmol), and 100 mL of distilled water. The mixture was heated up to 75 °C and stirred for 12 h. After cooling, the reaction mixture was filtered and neutralized with HCl. The resulting solution was extracted with diethyl ether. The combined organic fraction was washed with brine, dried over MgSO_4_, and concentrated by a rotary evaporator. The product was dried in vacuo at 90 °C for 14 h to give white powder **4** (2.53 g, 100.0% yield). ^1^H NMR (DMSO-*d*_6_, 400 MHz, ppm): 13.99 (broad, COOH, 3H), 8.09 (s, 1H), 8.05 (s, 1H). ^13^C NMR (DMSO-*d*_6_, 101 MHz, ppm): 167.06, 166.58, 166.49, 136.96, 134.68, 134.37, 129.67, 128.67, 128.34, 128.01, 127.68, 126.93, 126.82, 124.10, 121.38, 118.66.

**1,3-dioxo-6-(trifluoromethyl)-1,3-dihydroisobenzofuran-5-carboxylic acid (5)**: A 50 mL two-neck round-bottom flask equipped with a condenser was charged with **4** (2.49 g, 8.95 mmol) and 12 mL of 1,4-dioxane. Acetic anhydride (1.19 g, 1.08 mmol) was added to the reaction mixture, and the mixture was refluxed at 120 °C for 5 h. The mixture was poured into 300 mL of hexane and subsequently boiled with the addition of diethyl ether until the solution became clear. The solution was hot-filtered to remove any insoluble portion and gradually cooled to r.t. The solution was kept overnight in a refrigerator, and the recrystallized product was retrieved. The obtained product was dried overnight in vacuo at 80 °C. The product was purified via vacuum sublimation at 120 °C to give white powder **5** (1.52 g, 65.3% yield). ^1^H NMR (DMSO-*d*_6_, 400 MHz, ppm): 14.48 (broad, COOH, 1H), 8.53 (t, J = 0.6 Hz, 1H), 8.43 (t, J = 0.7 Hz, 1H).

2,2′-(2,2′-bis(trifluoromethyl)-[1,1′-biphenyl]-4,4′-diyl)bis(1,3-dioxo-6-(trifluoromethyl)isoindoline-5-carboxylic acid) (6): A 50 mL three-neck round-bottom flask with a nitrogen inlet was charged with *s*DA (826.24 mg, 2.58 mmol), 5 (1377.67 mg, 5.30 mmol), and 19 mL of DMAc. The mixture was stirred for 3.5 h at room temperature in an argon atmosphere. Acetic anhydride (2.37 g, 23.22 mmol) and DABCO (2.61 g, 23.22 mmol) were added to the reaction mixture, and the mixture was stirred for 14 h at room temperature. The mixture was poured into acidified water (pH 1). The resulting precipitate was subsequently washed with acidified water and distilled water. The solid was collected and dried in vacuo at 100 °C. The solid was re-dissolved in a methanol/acetone mixture and precipitated again in acidified water (pH 1). The resulting precipitate was collected and dried in vacuo at 100 °C and 180 °C to obtain white powder 6 (2.08 g, 100% yield). ^1^H NMR (DMSO-*d*_6_, 400 MHz, ppm): 14.39 (broad, COOH, 2H), 8.42 (d, J = 0.7 Hz, 2H), 8.36 (t, J = 0.7 Hz, 2H), 8.07 (d, J = 2.1 Hz, 2H), 7.89 (dd, J = 8.3, 2.1 Hz, 2H), 7.74 (d, J = 8.0 Hz, 2H).

**2-(4-carboxyphenyl)-1,3-dioxo-6-(trifluoromethyl)isoindoline-5-carboxylic acid (11)**: A 50 mL three-neck round-bottom flask with a nitrogen inlet was charged with 4-aminobenzoic acid (610.27 mg, 4.45 mmol), **5** (1126.02 mg, 4.33 mmol), and 17 mL of DMAc. The mixture was stirred for 3.5 h at room temperature in an argon atmosphere. Acetic anhydride (1.19 g, 11.61 mmol) and DABCO (1.30 g, 11.61 mmol) were added to the reaction mixture, and the mixture was stirred for 14 h at room temperature. The mixture was poured into acidified water (pH 1). The resulting precipitate was subsequently washed with acidified water and distilled water. The solid was collected and dried in vacuo at 100 °C. The solid was re-dissolved in a methanol/acetone mixture and precipitated again in acidified water (pH 1). The resulting precipitate was collected and dried in vacuo at 100 °C and 180 °C to obtain white powder **6** (1.64 g, 100% yield). ^1^H NMR (DMSO-*d*_6_, 400 MHz, ppm): 13.72 (broad, COOH, 2H), 8.35 (s, 1H), 8.30 (t, J = 0.7 Hz, 1H), 8.16–8.08 (m, 2H), 7.66–7.58 (m, 2H). ^13^C NMR (DMSO-*d*_6_, 101 MHz, ppm): 166.73, 166.71, 165.01, 138.87, 138.85, 135.45, 135.21, 133.35, 131.97, 131.65, 131.32, 130.99, 130.40, 130.01, 126.94, 126.81, 123.65, 121.79, 121.74, 121.69, 121.64, 121.36, 118.63.

### 2.4. Model Reaction

**Model Compound (8)**: A 25 mL three-neck round-bottom flask equipped with a nitrogen inlet was charged with **6** (321.78 mg, 0.4 mmol), 3-aminobenzotrifluoride (128.90 mg, 0.8 mmol), NMP (4 mL), pyridine (0.7 mL), triphenyl phosphite (0.7 mL), and CaCl_2_ (0.20 g). The reaction mixture was then heated to 100 °C and stirred for 6 h. During the reaction, small aliquots were taken from the mixture periodically and subjected to ^1^H NMR analysis to monitor the extent of the reaction. After completion, the mixture was cooled to room temperature and extracted with ethyl acetate and water. The organic layer was dried under MgSO_4_ and then evaporated by a rotary evaporator. The crude product was purified via silica gel column chromatography using ethyl acetate/hexane (*v*/*v* = 1:1.5) as an eluent. The product was dried in vacuo at 60 °C for 12 h. ^1^H NMR (DMSO-*d*_6_, 400 MHz, ppm): 11.24 (s, 2H), 8.52 (q, J = 0.7 Hz, 2H), 8.48 (d, J = 0.6 Hz, 2H), 8.23–8.18 (m, 2H), 8.14 (d, J = 2.2 Hz, 2H), 7.96 (dd, J = 8.2, 2.1 Hz, 2H), 7.94–7.91 (m, 2H), 7.78–7.73 (m, 2H), 7.71–7.62 (m, 2H), 7.54 (ddt, J = 7.8, 1.8, 0.9 Hz, 2H).

**Model Compound (9)**: The same procedure used for model compound **8** was repeated with ***s*DAc** (267.38 mg, 0.4 mmol), 3-aminobenzotrifluoride (128.90 mg, 0.8 mmol), NMP (4 mL), pyridine (0.7 mL), triphenyl phosphite (0.7 mL), and CaCl_2_ (0.20 g). The used eluent for the column chromatography was ethyl acetate/hexane (*v*/*v* = 1:1.2). ^1^H NMR (DMSO-*d*_6_, 400 MHz, ppm): 10.93 (s, 2H), 8.62 (dd, J = 1.5, 0.8 Hz, 2H), 8.50 (dd, J = 7.8, 1.5 Hz, 2H), 8.28 (dt, J = 2.4, 1.0 Hz, 2H), 8.20 (dd, J = 7.8, 0.7 Hz, 2H), 8.15–8.07 (m, 4H), 7.91 (dd, J = 8.2, 2.1 Hz, 2H), 7.71 (d, J = 8.3 Hz, 2H), 7.68–7.60 (m, 2H), 7.51 (ddt, J = 7.8, 1.9, 0.9 Hz, 2H).

**Model Compound (10)**: The same procedure used for model compound **8** was repeated with **6** (321.78 mg, 0.4 mmol), *p*-anisidine (98.52 mg, 0.8 mmol), NMP (4 mL), pyridine (0.7 mL), triphenyl phosphite (0.7 mL), and CaCl_2_ (0.20 g). The used eluent for the column chromatography was ethyl acetate/hexane (*v*/*v* = 1:1). ^1^H NMR (DMSO-*d*_6_, 400 MHz, ppm): 10.72 (s, 2H), 8.45 (s, 1H), 8.38 (s, 1H), 8.12 (d, J = 2.1 Hz, 1H), 7.94 (dd, J = 8.2, 2.1 Hz, 1H), 7.75 (d, J = 8.4 Hz, 1H), 7.66–7.59 (m, 2H), 7.01–6.94 (m, 2H), 3.77 (s, 6H).

### 2.5. Polymerization

**PAI (7)**: A 25 mL three-neck round-bottom flask equipped with a mechanical stirrer and a nitrogen inlet was charged with a mixture of ***s*DA** (192.14 mg, 0.60 mmol), **6** (482.67 mg, 0.60 mmol), NMP (5 mL), pyridine (1 mL), triphenyl phosphite (1 mL), and CaCl_2_ (0.30 g). The reaction was carried out at 100 °C under an argon atmosphere for 8 h. As the polycondensation proceeded, the reaction mixture became gradually viscous, and a small amount of additional NMP was added periodically (total addition volume = 5 mL) during the polymerization to maintain a proper viscosity. At the end of the reaction, the polymer solution was cooled to room temperature and poured slowly into an excess amount of vigorously stirred methanol/water mixture (*v*/*v* = 1:1). The precipitate was washed thoroughly with methanol/H_2_O mixture and then dried in vacuo at 100 °C for 12 h. The product was re-dissolved in DMAc and precipitated again in a methanol/H_2_O (*v*/*v* = 1:1) mixture. The resulting precipitate was collected and dried in vacuo at 180 °C for 18 h (629.21 mg, 96.3% yield). ^1^H NMR (DMSO-*d*_6_, 400 MHz, ppm): 11.35 (s, 2H), 8.58 (s, 2H), 8.51–8.50 (m, 2H), 8.31 (s, 2H), 8.13 (s, 2H), 8.08 (t, J = 2.7 Hz, 2H), 7.99–7.95 (m, 2H), 7.76 (t, J = 7.1 Hz, 2H), 7.49–7.43 (m, 2H).

**CF_3_-PAI 1**: The same protocol was repeated with ***s*DA** (288.21 mg, 0.90 mmol) and **11** (341.32 mg, 0.90 mmol). During the polymerization, 2 mL of NMP was added (552.94 mg, 92.6% yield). FT-IR (film, cm^−1^): 3298 (amide N-H stretching); 1787, 1728 (imide C=O); 1670 (amide C=O); 1529 (amide N-H bending); 1489, 1417 (aromatic C=C); 1379 (imide C-N stretching); 1169, 1117 (C-F in CF_3_); 723 (imide ring deformation). ^1^H NMR (DMSO-*d*_6_, 400 MHz, ppm): 11.33 (s, 1H), 10.80 (s, 1H), 8.54 (s, 1H), 8.47 (s, 1H), 8.40 (s, 1H), 8.29 (s, 1H), 8.22–8.09 (m, 3H), 7.99 (d, J = 8.9 Hz, 1H), 7.80–7.64 (m, 3H), 7.52–7.34 (m, 1H).

**CF_3_-PAI 2**: The same protocol was repeated with ***s*DA** (288.21 mg, 0.90 mmol), **TA** (14.95 mg, 0.09 mmol), and **11** (307.18 mg, 0.81 mmol). During the polymerization, 5 mL of NMP was added (549.62 mg, 95.1% yield). FT-IR (film, cm^−1^): 3298 (amide N-H stretching); 1787, 1728 (imide C=O); 1666 (amide C=O); 1529 (amide N-H bending); 1489, 1417 (aromatic C=C); 1379 (imide C-N stretching); 1169, 1117 (C-F in CF_3_); 723 (imide ring deformation). ^1^H NMR (DMSO-*d*_6_, 400 MHz, ppm): 11.33 (s, 0.9H), 10.83 (s, 0.2H), 10.79 (s, 0.9H), 8.54 (s, 0.9H), 8.47 (s, 0.9H), 8.40 (s, 1.1H), 8.29 (s, 0.9H), 8.18 (m, 3.1H), 7.99 (d, J = 8.8 Hz, 0.9H), 7.80–7.64 (m, 2.7H), 7.52–7.34 (m, 1.1H).

**CF_3_-PAI 3**: The same protocol was repeated with ***s*DA** (320.23 mg, 1.00 mmol), **TA** (49.84 mg, 0.30 mmol), and **11** (265.47 mg, 0.70 mmol). During the polymerization, 7 mL of NMP was added (567.77 mg, 94.7% yield). FT-IR (film, cm^−1^): 3302 (amide N-H stretching); 1787, 1728 (imide C=O); 1664 (amide C=O); 1529 (amide N-H bending); 1489, 1417 (aromatic C=C); 1379 (imide C-N stretching); 1169, 1117 (C-F in CF_3_); 723 (imide ring deformation). ^1^H NMR (DMSO-*d*_6_, 400 MHz, ppm): 11.33 (s, 0.7H), 10.84 (s, 0.6H), 10.80 (s, 0.7H), 8.54 (s, 0.7H), 8.47 (s, 0.7H), 8.40 (s, 1.3H), 8.29 (s, 0.7H), 8.18 (m, 3.3H), 7.99 (d, J = 9.2 Hz, 0.7H), 7.80–7.64 (m, 2.1H), 7.52–7.34 (m, 1.3H).

**CF_3_-PAI 4**: The same protocol was repeated with ***s*DA** (352.25 mg, 1.10 mmol), **TA** (91.37 mg, 0.55 mmol), and **11** (208.58 mg, 0.55 mmol). During the polymerization, 8 mL of NMP was added (590.55 mg, 96.4% yield). FT-IR (film, cm^−1^): 3302 (amide N-H stretching); 1787, 1728 (imide C=O); 1662 (amide C=O); 1522 (amide N-H bending); 1489, 1417 (aromatic C=C); 1379 (imide C-N stretching); 1169, 1117 (C-F in CF_3_); 723 (imide ring deformation). ^1^H NMR (DMSO-*d*_6_, 400 MHz, ppm): 11.32 (s, 0.5H), 10.83 (s, 1H), 10.80 (s, 0.5H), 8.54 (s, 0.5H), 8.47 (s, 0.5H), 8.40 (s, 1.5H), 8.30 (s, 0.5H), 8.24–8.11 (m, 3.5H), 8.02–7.94 (m, 0.5H), 7.80–7.64 (m, 1.5H), 7.52–7.34 (m, 1.5H).

### 2.6. Preparation of Poly(amide-imide)s Films

Films were prepared by dissolving 0.15 g of PAIs in 4 mL of *N,N*-dimethylacetamide (DMAc) to prepare a 3.75 wt% polymer solution at room temperature. The solution was passed through a PTFE syringe filter (5.0 μm) and cast on a 5 × 5 cm glass plate. The solvent was slowly evaporated in a vacuum oven at 35 °C for 4 h and 180 °C overnight to eradicate residual solvents.

## 3. Results and Discussion

### 3.1. Monomer Synthesis

**Scheme 1:**
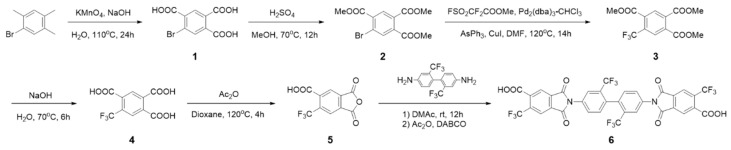
Synthetic scheme of the trifluoromethylated trimellitic anhydride and the diacid monomer.

The trifluoromethylated trimellitic anhydride (**5**) and diacid monomer (**6**) with diimide moiety were synthesized as shown in Scheme 1. Oxidation of 5-bromo-1,2,4-trimethylbenzene compound **1** was carried out with KMnO_4_. Oxidation of the methyl groups to carboxylic acid was carried out before trifluoromethylation of 5-bromo-1,2,4-trimethylbenzene because oxidation of trifluoromethylated pseudocumene did not proceed well. The carboxylic acid groups were protected by converting to ester groups (**2**) by an acid-catalyzed esterification reaction because of catalyst poisoning during the trifluoromethylation reaction.

**Figure 1:**
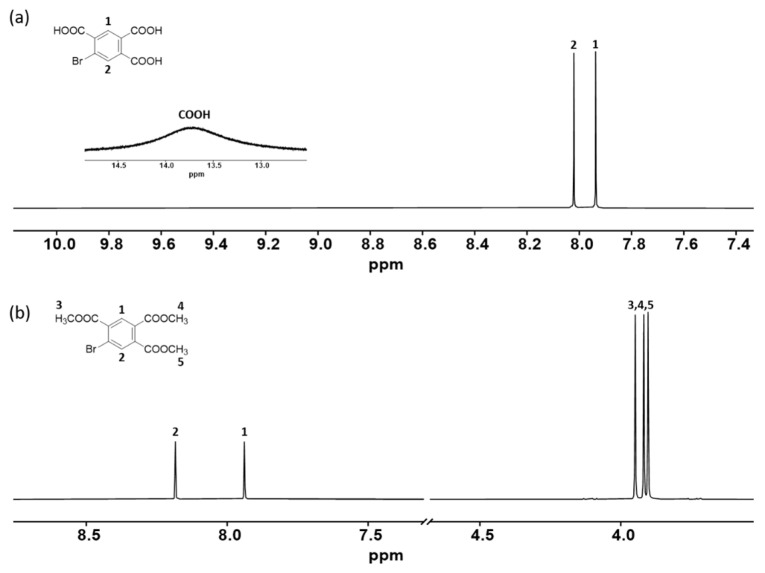
^1^H NMR spectra of (**a**) **1** (DMSO-*d*_6_) and (**b**) **2** (Chloroform-*d*).

**Figure 2:**
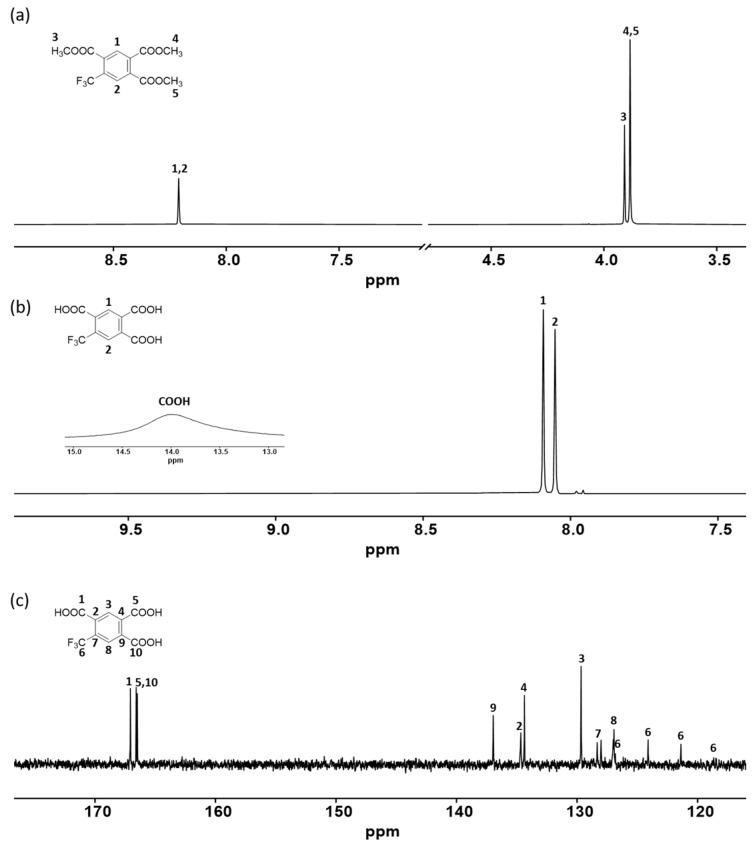
^1^H NMR spectra of (**a**) **3** and (**b**) **4** (DMSO-*d*_6_), and ^13^C NMR spectrum of (**c**) **4** (DMSO-*d*_6_).

The NMR analysis of the synthesized compounds was carried out in deuterated solvents, and the chemical shift in the NMR signals was reported in parts per million (ppm) using tetramethylsilane (TMS) as an internal reference. NMR analyses of **1** (Figure 1a) and **2** (Figure 1b) revealed that the oxidation and esterification reactions proceeded successfully. Trifluoromethylation of **2** with methyl difluoro(fluorosulfonyl)acetate (FSO_2_CF_2_COOMe) and Pd catalyst (Pd_2_(dba)_3_-CHCl_3_) produced the trifluoromethylated triester compound **3** quantitatively (Figure 2a). Trifluoromethylated trimellitic acid (**4**) was obtained by subsequent hydrolysis using NaOH (Figure 2b,c).

**Figure 3:**
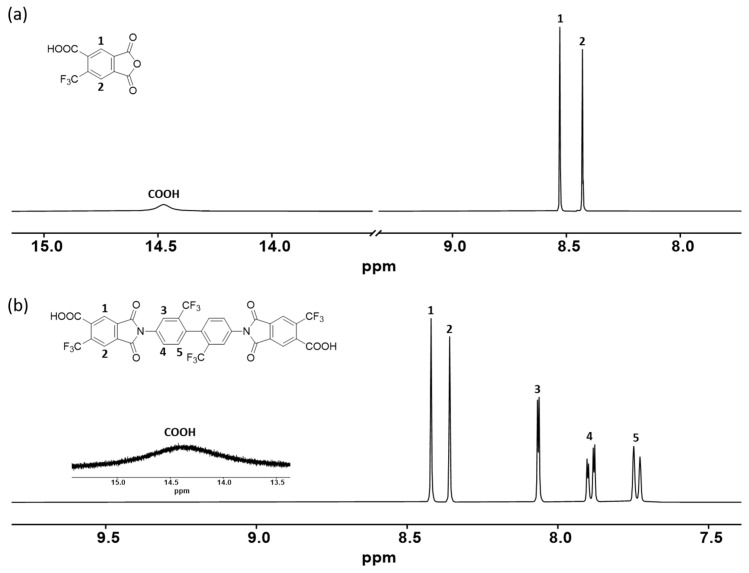
^1^H NMR spectra of (**a**) **5** and (**b**) **6** (DMSO-*d*_6_).

Cyclodehydration of **4** to obtain trifluoromethylated trimellitic anhydride (**5**) was carried out with 1,4-dioxane and acetic anhydride. Compound **5** was purified by recrystallization in hexane and diethyl ether followed by vacuum sublimation at 120 °C (Figure 3a). During this purification step, the small amount of side products in **4** (Figure 2b) were removed. Finally, the reaction of **5** with 2,2′-bis(trifluoromethyl)benzidine (***s*DA**) and the subsequent cyclodehydration in acetic anhydride produced the diacid monomer **6** containing imide rings (Figure 3b).

**Scheme 2:**
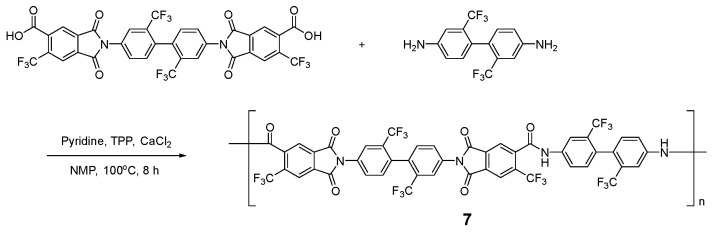
Polymerization of the diacid monomer **6** with ***s*DA**.

Polymerization of the diacid monomer **6** with diamine monomer ***s*DA** was attempted to make poly(amide-imide) (**7**) via the Yamazaki–Higashi polycondensation method (Scheme 2) [40]. However, as the polymerization proceeded, the viscosity of the polymer increased significantly, and gelation occurred. Analysis of the soluble fraction of the polymerization by ^1^H NMR revealed several peaks corresponding to amide bonds and terminal amine groups. The polymerization process did not seem to proceed as expected, and we carried out model reactions to investigate the reaction behavior of the trifluoromethylated trimellitic monomer (**6**).

### 3.2. Model Reaction

**Scheme 3:**
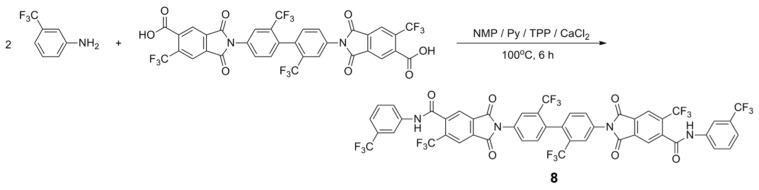
Model reaction of **6** with 3-aminobenzotrifluoride.

**Figure 4:**
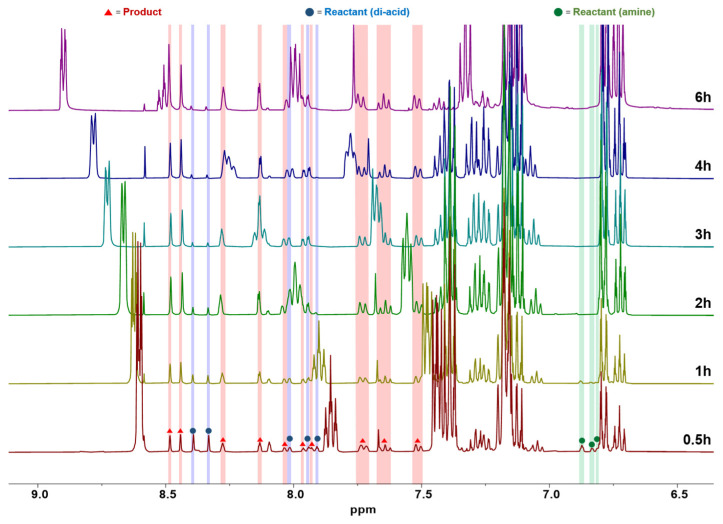
^1^H NMR spectra of model reaction for **8**, aromatic region (DMSO-*d*_6_).

The first model reaction was carried out as shown in Scheme 3 using the synthesized diacid **6** and 3-aminobenzotrifluoride. The reaction condition was similar to the polymerization of poly(amide-imide) **7**, and the reaction was followed by ^1^H NMR analysis. As shown in Figure 4, in the aromatic region, the amine reactant was completely consumed after 2 h, while the diacid reactant persisted until the end of the reaction. Because a stoichiometric amount of the diacid monomer and amine was used, the above results clearly indicate that the amine reactant reacted not only with the carboxylic acid but also with another functional group in the diacid monomer.

**Figure 5:**
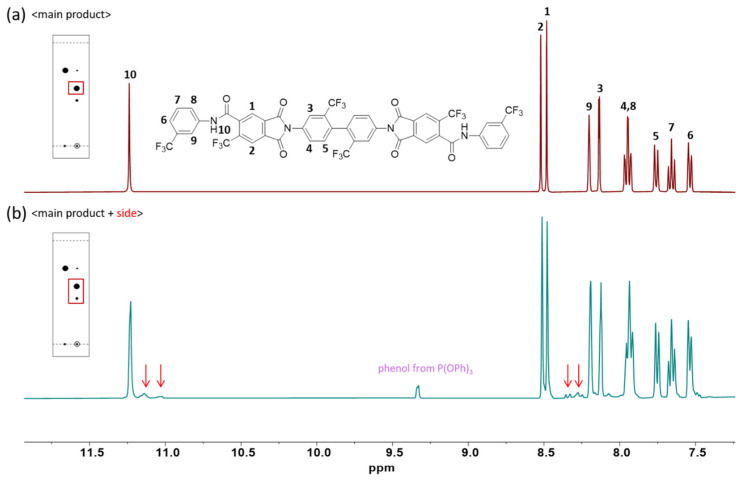
^1^H NMR spectra of (**a**) model compound **8** and (**b**) **8** with side products (DMSO-*d*_6_).

The main product of the model reaction of **6** with 3-aminobenzotrifluoride was separated by silica gel column chromatography and further analyzed by ^1^H NMR spectroscopy (Figure 5a). As expected, the main product was the model compound **8**, but there were side products in the reaction product (Figure 5b, red arrows). The attempt to separate this side product was unsuccessful due to its small amount, but two different types of side amide peaks were observed in the amide region of the NMR spectrum (main product + side product).

**Scheme 4:**
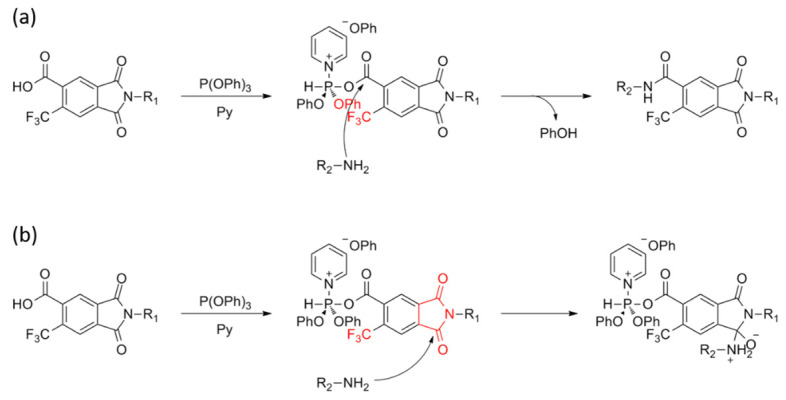
Scheme of the proposed model (**a**) and side (**b**) reactions (highlighted in red color).

In this model reaction, we observed (1) a relatively slow conversion of diacid monomer, (2) the formation of side products, and (3) the presence of diacid monomer **6** after the complete consumption of the amine reactant. These model reaction results suggest that the electron-withdrawing CF_3_ group attached to the trimellitic structure gives rise to the imide ring susceptible to nucleophilic attack, resulting in the reactant amine attack of the imide ring (Scheme 4).

**Scheme 5:**
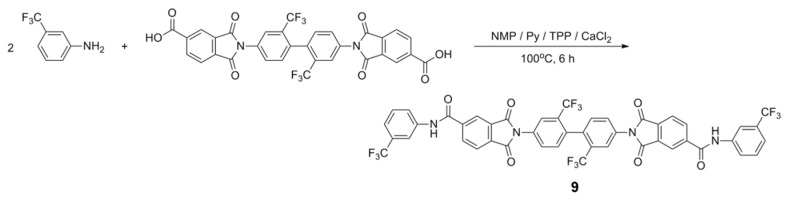
Model reaction of ***s*DAc** with 3-aminobenzotrifluoride.

**Figure 6:**
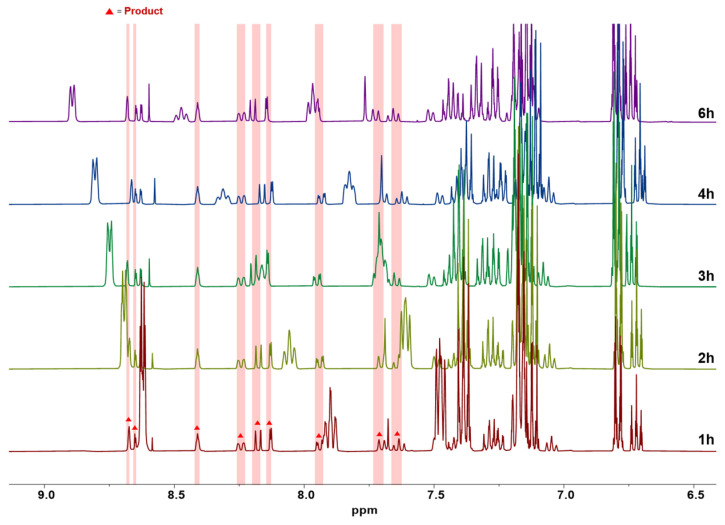
^1^H NMR spectra of model reaction for **9**, aromatic region (DMSO-*d*_6_).

**Figure 7:**
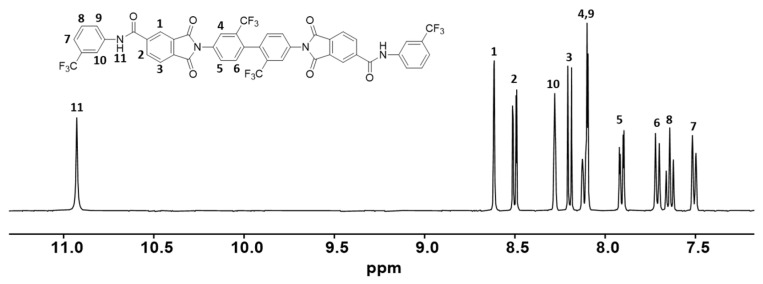
^1^H NMR spectrum of the model compound **9** (DMSO-*d*_6_).

To confirm the effect of the trifluoromethyl group in the trimellitic unit on the proposed side reaction, the second model reaction (Scheme 5) was carried out with the diacid compound having a trimellitic unit without a trifluoromethyl group, 2,2′-bis(trifluoromethyl)-4,4′-bis(trimellitimido)biphenyl (***s*DAc**). The same reaction condition was used as the previous model reaction, and the model reaction was followed by ^1^H NMR analysis (Figure 6). ^1^H NMR spectra analysis revealed that the model reaction with ***s*DAc** proceeded quite differently. The diacid and amine compounds disappeared quickly, and both amine and diacid compounds were completely consumed within 1 h (Figure 6). The ^1^H NMR of the product indicates that there were no other side products (Figure 7).

**Scheme 6:**
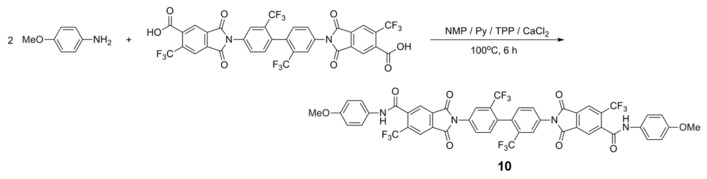
Model reaction of **6** with *p*-anisidine.

**Figure 8:**
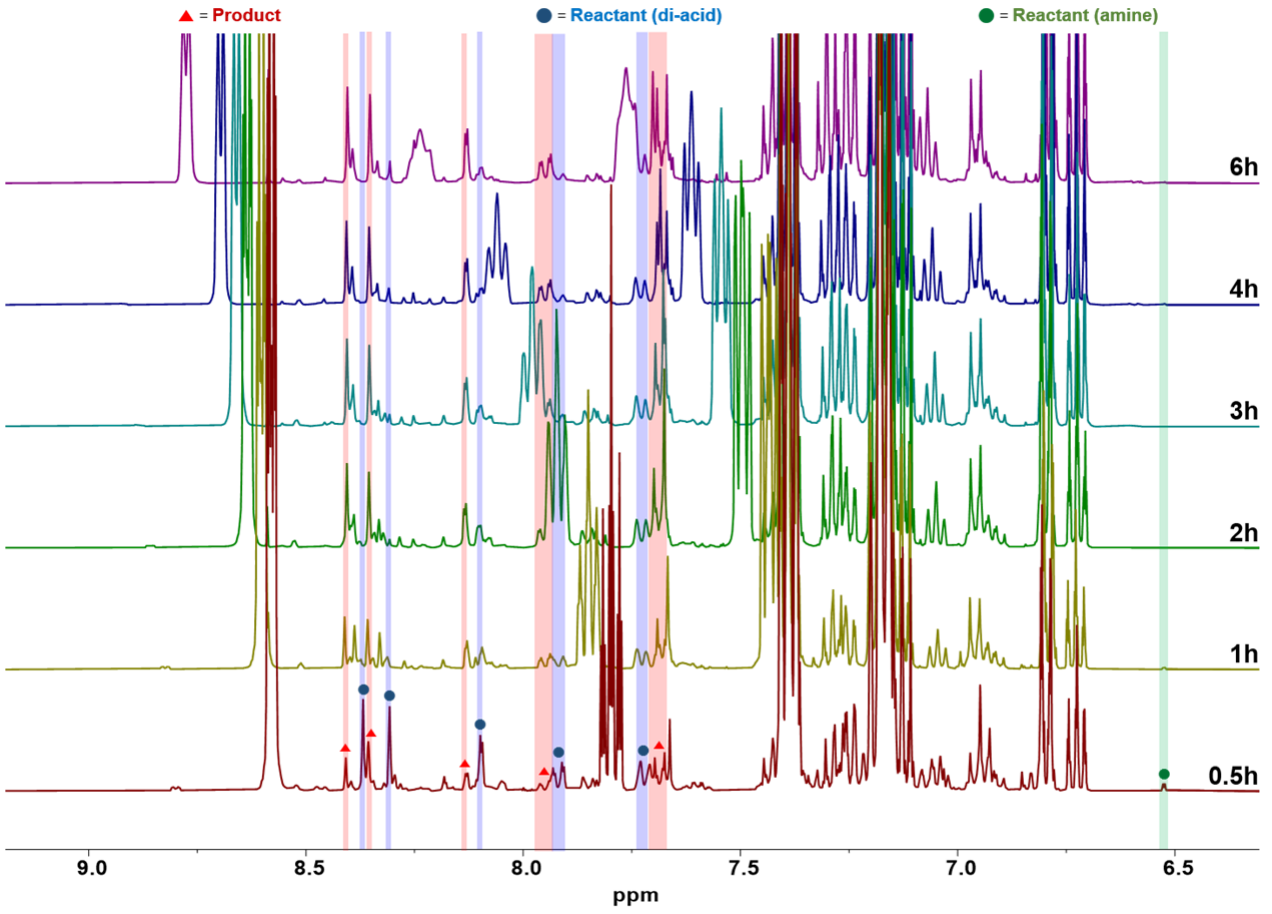
^1^H NMR spectra of model reaction for **10**, aromatic region (DMSO-*d*_6_).

**Figure 9:**
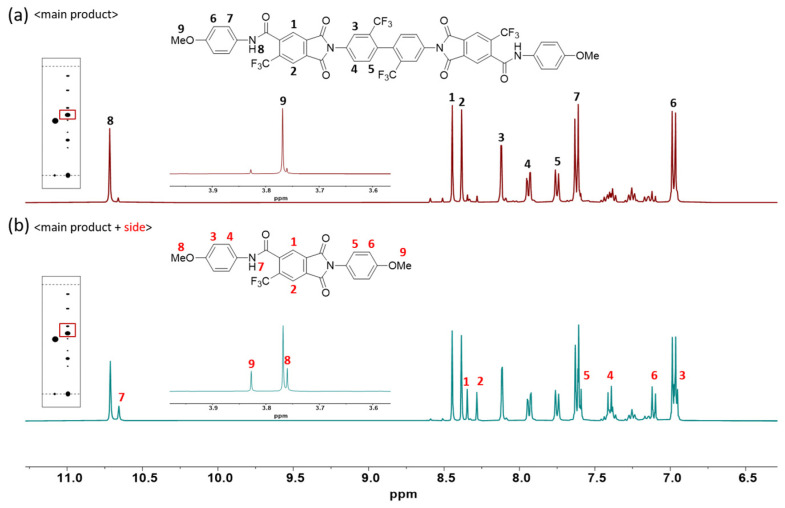
^1^H NMR spectra of (**a**) model compound **10** and (**b**) **10** with side products (DMSO-*d*_6_).

To check the effect of the nucleophiles, the third model reaction was carried out with a more reactive amine, *p*-anisidine, as shown in Scheme 6. The same reaction condition was used as the previous model reaction, and the model reaction was followed by ^1^H NMR analysis (Figure 8). As shown in Figure 8, in the aromatic region, the amine reactant was completely consumed after 1 h, while the diacid reactant persisted until the end of the reaction. The reaction proceeded similarly to model reaction 1, but more rapidly, presumably because *p*-anisidine is more reactive than 3-aminobenzotrifluoride. ^1^H NMR of the product shows an increase in side products (Figure 9), indicating that the side reaction was facilitated as the nucleophilicity of the amine increased.

We attempted to separate the product of the model reaction from the by-product, but it was unsuccessful. Instead of the clear separation of the main product by column chromatography, we prepared two NMR samples: one with a leading product content of 90% or higher and the other with one of several side products of 20% or higher. ^1^H NMR analysis of the two samples revealed the structure of the side product, as shown in Figure 9.

**Scheme 7:**
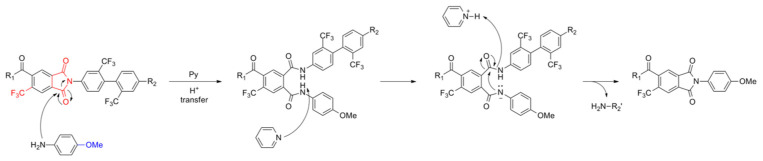
Proposed mechanism of side reaction in the model reaction for **10** (highlighted in red color).

The presence of this side product is strong evidence that a nucleophilic attack on the imide ring occurred. Based on the results of the above three model reactions, the mechanism of the side reaction in the model reaction is proposed in Scheme 7. The nucleophilic attack of amine first opens the imide ring and then leads to deprotonation by pyridine, followed by the nucleophilic attack of the amide anion, giving rise to substituting the imide bond, known as trans-imidization. This trans-imidization reaction was not observed in the model reaction with ***s*DAc**, suggesting that it is caused by the CF_3_ group substituted into the trimellitic structure.

### 3.3. Modification of Monomer Structure

**Scheme 8:**
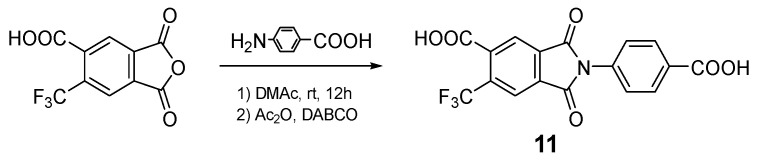
Synthesis of the diacid monomer **11**.

We modified the structure of diacid monomer **6** to avoid the side reaction observed in the model reaction by reducing the number of imide rings susceptible to nucleophiles and the number of CF_3_ substituents near the imide bond, as shown in Scheme 8. The trifluoromethylated trimellitic anhydride was reacted with 4-aminobenzoic acid and cyclodehydrated with acetic anhydride.

**Figure 10:**
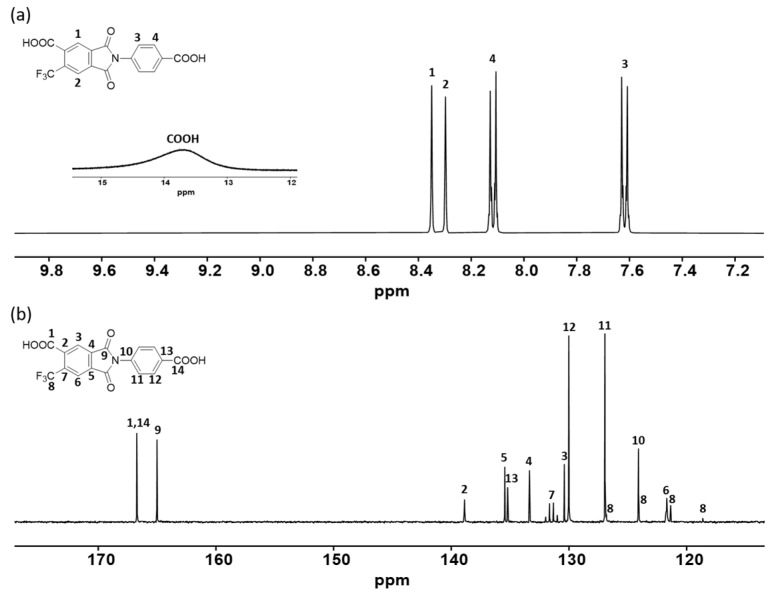
(**a**) ^1^H NMR spectrum and (**b**) ^13^C NMR spectrum of **11** (DMSO-*d*_6_).

^1^H NMR and ^13^C NMR spectra of the diacid monomer 11 (Figure 10) show the proton peaks corresponding to the monomer structure, and the proton peak of the amic acid group is absent, indicating the successful synthesis of the diacid monomer 11. In the ^13^C NMR spectrum, the unique splitting pattern by C-F coupling is observed in the carbon numbers 8 and 7.

### 3.4. Polymerization

**Scheme 9:**
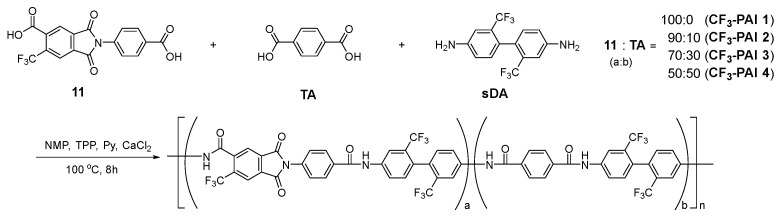
Polymerization of the diacid monomer **11**.

A series of poly(amide-imide)s (PAIs) were synthesized from the diacid monomer **11**, terephthalic acid (**TA**) monomer, and ***s*DA** diamine monomer via the Yamazaki–Higashi polycondensation method (Scheme 9). Polymerization was achieved by reacting the diamine monomer with a stoichiometric amount of two dicarboxylic acid monomers in NMP, with TPP and pyridine as condensing agents. The polymerizations were carried out for 8 h at 100 °C. The initial solid concentrations were around 10 *w*/*v*%, but as the polymerization progressed, the viscosity drastically rose. Thus, more NMP was added to the reaction solution to prevent gelation. White fibrous polymers were produced by precipitating the polymerization mixture into an excess methanol/water (*v*/*v* = 1:1) mixed solvent.

Table 1 summarizes the polymerization yields and inherent viscosities of the synthesized PAIs. The resulting PAIs exhibited inherent viscosities ranging from 1.57 to 2.90 dL/g, confirming that the molecular weights of all the PAIs were sufficiently high to cast flexible and robust films. Because the structural characteristics of the two diacid monomers differ, the variance in inherent viscosity is affected by the ratio of monomers employed. **TA** makes the polymer main chain more linear than diacid **11**, and the more **TA** is used, the more amide bonds are in the polymer chain. As the **TA** contents increase, so do the inherent viscosities.

**Figure 11:**
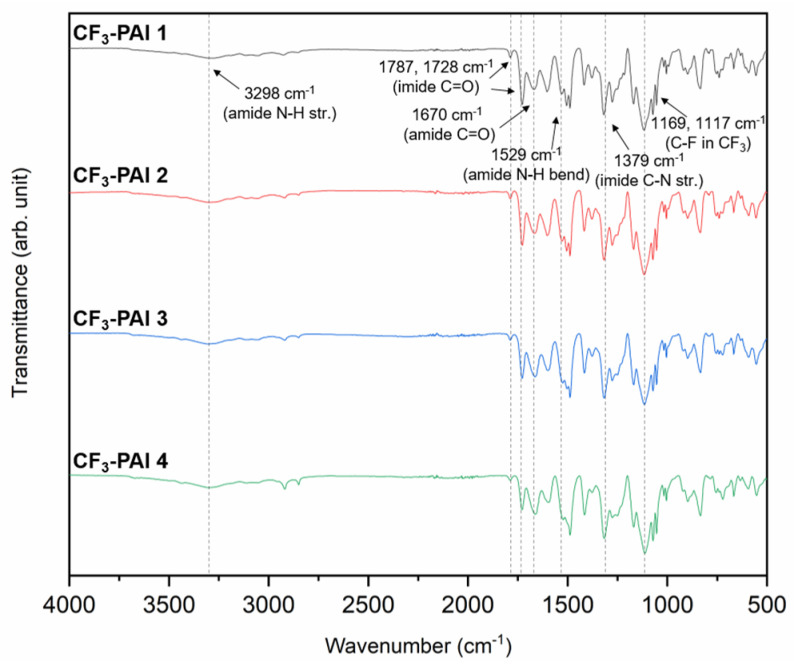
FT-IR spectra of PAIs (film).

**Figure 12:**
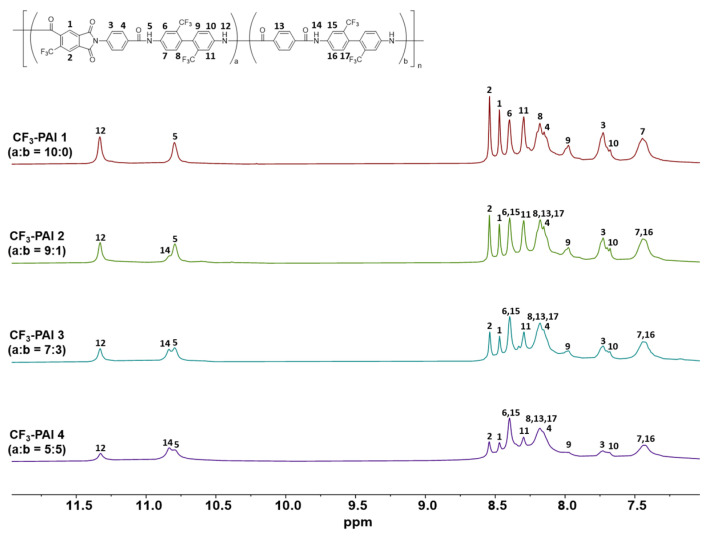
^1^H NMR spectra of PAIs (DMSO-*d*_6_).

FT-IR spectroscopy and ^1^H NMR spectroscopy were used to confirm the production and chemical structures of the PAIs. Figure 11 depicts the FT-IR spectra of PAIs. All PAIs displayed typical absorption bands corresponding to their functional groups at 3298 cm^−1^ (amide N-H); 1787 and 1728 cm^−1^ (imide C=O); 1670 cm^−1^ (amide C=O); 1379 cm^−1^ (imide C-N stretching); and 1169 and 1117 cm^−1^ (CF_3_ groups owing to C-F stretching). Figure 12 depicts the ^1^H NMR spectra of the PAIs. The proton peaks were correctly assigned to their expected structure, indicating that the PAIs were successfully prepared. We noticed that the peak intensities changed as the diacid monomer ratio varied. However, due to strong hydrogen bonding and structural similarities, calculating the exact ratio of two diacid monomers in the polymer backbone was not feasible. As hydrogen bonds increased, peak broadening became more severe.

### 3.5. Properties of Polymers

The solubility of the synthesized PAIs in various solvents was investigated, and the results are summarized in Table 2. At room temperature, all PAIs were soluble in polar aprotic solvents such as N-methyl-2-pyrrolidone, N,N-dimethylacetamide, and N,N-dimethylformamide. PAIs had improved solubility and were even soluble in DMSO, unlike ***s*DA-TA**. The improved solubility of PAIs is due to the presence of bulky CF3 groups on the polymer backbone and the unsymmetrical structure of diacid **11**, which causes distortion in polymer chains and decreases interchain interaction.

**Figure 13:**
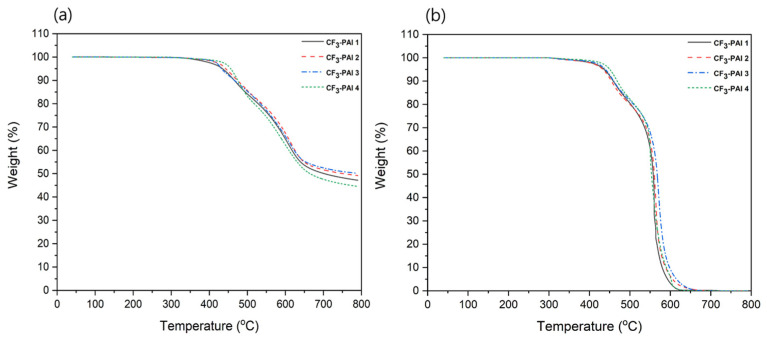
TGA curves of the PAIs in (**a**) N_2_ and (**b**) air at a heating rate of 10 °C/min.

TGA, DSC, and TMA were used to analyze the thermal characteristics of the PAIs, and the findings are reported in Table 3. TGA studies revealed that all of the synthesized PAIs had high thermal stability, with decomposition temperatures of 5% weight loss (*T*_d5_) ranging from 434 to 452 °C in N_2_ and 428 to 446 °C in air, respectively (Figure 13). The phenomenon of increasing *T*_d5_ when the TA ratio increases is related to the molecular structure of TA. Because two carboxyl groups are placed in the *para-*position, TA induces a rigid chain structure and increases chain alignment.

**Figure 14:**
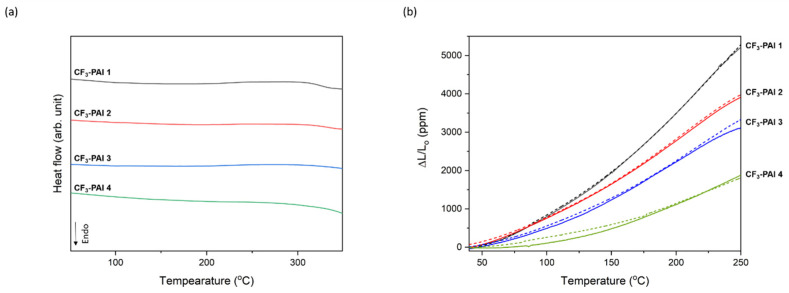
(**a**) DSC curves of the PAIs (the second heating run ranging from 0 °C to 350 °C at a heating rate of 10 °C/min in N_2_). (**b**) TMA curves of the PAIs (the second (solid) and third (dash) heating runs ranging from 40 °C to 250 °C at a heating rate of 5 °C/min in N_2_).

DSC experiments were used to examine the glass transition temperature of PAIs. The heat flow graphs of the second cycle, obtained through DSC measurement, are shown in Figure 14a. Endothermic signals were seen at 325 °C and 336 °C in **CF_3_-PAI 1** and **CF_3_-PAI 2**, respectively. On the other hand, in the case of **CF_3_-PAI 3** and **CF_3_-PAI 4,** endothermic signals were not observed below 350 °C. When the observation temperature exceeded 350 °C, thermal decomposition occurred. The glass transition temperature is expected to be proportional to the TA content because of the stiff structure and increased hydrogen bonding.

The thermodimensional stability of the PAI film was examined three times in a sequence using TMA while the temperature was raised from 40 °C to 250 °C (Figure 14b). The coefficient of thermal expansion (CTE) values were calculated after measuring the changes in the length of the films depending on the temperature rise, and values ranging from 9.0 to 26.6 ppm/°C were obtained in the third run. The CTE values ranging from 9 to 27 ppm/°C, together with high T_g_ values over 325 °C, clearly indicate that PAIs have much better thermal and dimensional stability compared to PET (T_g_: 80 °C; CTE: 33 ppm/°C) and PES (T_g_: 223 °C; CTE: 54 ppm/°C). The measurement findings revealed that as the fraction of TA increased, CTE lowered, and **CF_3_-PAI 4**, polymerized with the same amount of TA and **11**, had a very low CTE value of 10 ppm/°C. Because of the strong chain alignment toward the direction parallel to the film plane, rigid-rod polymers with more rectilinear structures generally have lower CTE values. Therefore, the polymer chain becomes stiffer as the TA content rises, lowering the CTE. Interestingly, it was found that **CF_3_-PAI 2**, **3**, and **4** showed lower CTE values compared to ***s*DA-TA**. This result suggests that the ratio of amide bonds to imide bonds influences the CTE of PAI.

**Figure 15:**
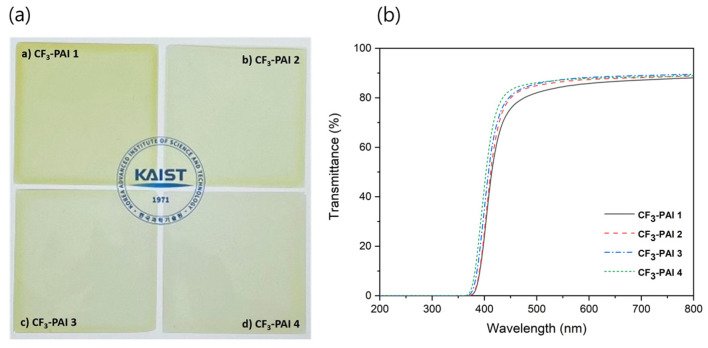
(**a**) Images of the PAI films. (**b**) Transmittance UV-Vis spectra of the PAI films.

By casting the DMAc solutions, tough and flexible films of PAIs were obtained (Figure 15). The optical transmittance of the films was measured by a UV-Vis spectrometer, and the results are shown in Figure 15b and Table 4. The transmission at 550 nm of PAIs is 87% comparable to PES (89%). These PAI films appear slightly yellow, but the cutoff wavelength is decreased (356–362 nm) compared to the PAIs made from ***s*DAc** (371 nm). Compared to the homopolymers (**CF_3_-PAI 1**), the PAI copolymers obtained by copolymerization with TA, **CF_3_-PAI 2**, **CF_3_-PAI 3**, and **CF3-PAI 4** showed improved optical properties. The bent and unsymmetrical structure of imide-diacid **11** seems to be the reason for this result. **CF_3_-PAIs** showed similar cutoff wavelengths from 356 nm to 362 nm, and the transmittance at 400 nm and 550 nm increased as the TA content increased. The more irregular structures of the repeating unit from the two components there are, the more effectively this suppresses the formation of the CT complex. Also, as the TA content increases, the proportion of imide bonds in the entire polymer chains decreases, reducing the formation of the CT complex and increasing the transparency of the PAI films.

## 4. Conclusions

A new trifluoromethylated trimellitic anhydride was synthesized and converted to a diacid monomer containing imide rings (**6** and **11**). The electron-withdrawing capability of CF_3_ groups makes the imide ring of the trimellitic structure susceptible to the nucleophilic attack of amines, resulting in side reactions, including ring opening and trans-imidization reactions. High-molecular-weight PAIs were synthesized from the diacid monomer with fewer CF_3_ groups (**11**). Polycondensation of the diacid monomer with diamines by the Yamazaki–Higashi polycondensation method produced trifluoromethylated poly(amideimide)s having high transparency (84~87% transmittance at 550 nm) and good thermodimensional stability (CTE values from 9 to 26 ppm/°C). The PAIs showed good solubility in polar aprotic solvents. Their *T*_d5_ and *T*_g_ increased as the TA content increased, but CTE decreased because of the rigid structure and the hydrogen bonds of the amide and CF_3_ groups. Transparent PAIs with high thermal and thermodimensional stability may find applications in developing transparent and flexible electronics.

## Figures and Tables

**Table 1:** Polymerization yield and inherent viscosity of the PAIs.

Polymer Code	Yield (%)	Inherent Viscosity (η_inh_) ^1^ (dL/g)
**CF_3_-PAI 1**	92.6	1.57
**CF3-PAI 2**	95.1	1.86
**CF3-PAI 3**	94.7	1.75
**CF3-PAI 4**	96.4	2.90
**sDA-TA 1** ^2^	99.9	4.39

^1^ Measured in DMAc at a concentration of 0.5 g/dL at 30 °C. Each sample was measured 5 times and averaged. ^2^ Obtained from ref. [41].

**Table 2:** Solubility of the PAIs ^a^.

Solvents	CF_3_-PAI 1	CF_3_-PAI 2	CF_3_-PAI 3	CF_3_-PAI 4
NMP	++	++	++	++
DMAc	++	++	++	++
DMF	++	++	++	++
DMSO	++	++	++	+
*m*-cresol	−s	−s	−s	+
THF	++	+	−s	−s
Acetone	−s	−s	−s	−s
Ethyl acetate	−	−	−	−

^a^ Solubility: ++, soluble at room temperature; +, soluble on heating; −s, swelling; −, insoluble. Abbreviations: NMP, *N*-methyl-2-pyrrolidone; DMAc, *N*,*N*-dimethylacetamide; DMF, *N*,*N*-dimethylformamide; DMSO, dimethyl sulfoxide; THF, tetrahydrofuran.

**Table 3:** Thermal properties of the PAIs.

Polymer Code	*T*_g_ (°C) ^1^	*T*_d5_ (°C) ^2^		CTE (ppm/°C) ^3^	
		In N_2_	In Air	2nd Run	3rd Run
**CF_3_-PAI 1**	325	437	428	26.7	26.6
**CF_3_-PAI 2**	336	445	434	19.9	19.7
**CF_3_-PAI 3**	-	434	440	16.6	16.8
**CF_3_-PAI 4**	-	452	446	9.8	9.0

^1^ Measured by DSC (the second scan) in N_2_ at a heating rate of 10 °C/min; ^2^ 5% weight loss temperature, measured by TGA at a heating rate of 10 °C/min. ^3^ Coefficient of thermal expansion, measured by TMA at a temperature range from 40 to 250 °C at a heating rate of 5 °C/min in N_2_.

**Table 4:** Optical properties of the PAIs.

Polymer Code	Cutoff Wavelength (nm)	Transmittance at 400 nm (%)	Transmittance at 550 nm (%)	Film Thickness (μm)
**CF_3_-PAI 1**	362	24.2	84.5	30
**CF_3_-PAI 2**	361	26.2	86.5	30
**CF_3_-PAI 3**	358	35.3	87.4	30
**CF_3_-PAI 4**	356	44.2	87.2	30

## Data Availability

The original contributions presented in this study are included in the article. Further inquiries can be directed to the corresponding author.
